# High prevalence of dental fluorosis among adolescents is a growing concern: a school based cross-sectional study from Southern India

**DOI:** 10.1186/s12199-017-0624-9

**Published:** 2017-04-04

**Authors:** Anand Verma, Bharatesh K. Shetty, Vasudeva Guddattu, Mehul K. Chourasia, Prachi Pundir

**Affiliations:** 1 Directorate of Health Services, Naya Raipur, Chhattisgarh India; 2 OVC Special Protection Project, Karnataka Health Promotion Trust, Pune Maharashtra, India; 3 Department of Statistics, Manipal University, Manipal, India; 4 National Institute of Malaria Research (NIMR), ICMR, Kondagaon, Chhattisgarh India; 5 IPE Global Ltd, New Delhi, India; 6 IPE Global House, B-84, Defence colony, New Delhi, 110024 India

**Keywords:** Adolescents, Community Fluorosis Index, Dental fluorosis, Dean’s Fluorosis Index

## Abstract

**Background:**

Fluorosis, caused by ingestion of excessive amount of fluoride through food or water, is a major public health problem in India. This study was undertaken to quantify the dental fluorosis burden among school going adolescents and to find factors associated with dental fluorosis in Kolar taluka, Karnataka, India.

**Methods:**

A total of 1026 high school adolescents (12–17 years) were enrolled from different schools selected by stratified sampling method. Dental examination was done to record Dean’s fluorosis index, and socio-demographic, food consumption and oral hygiene data were recorded using a pre-tested structured questionnaire. Fluoride content was measured using Orion apparatus, and Community Fluorosis Index (CFI) was calculated from drinking water samples from various drinking sources. Multivariable analysis with generalized estimating equation (GEE) regression model was used to explore the factors associated with dental fluorosis.

**Result:**

Among 1026 enrolled students, 64.3% of adolescents were detected with dental fluorosis; more than 50% had either severe or moderate fluorosis according to the Dean’s Fluorosis Index and Community Fluorosis Index (CFI). The majority of affected students were from government schools. The significantly associated factors with dental fluorosis were living in study area for more than 5 years and studying in government school. A strong positive correlation between the amount of fluoride content in drinking water sample collected and CFI was observed (rho = 0.570).

**Conclusion:**

Prevalence of dental fluorosis was considerably high, affecting nearly two-thirds of the students, and mainly in government schools and long-term residents of the area. Health education and community awareness for preventing fluorosis, apart from setting-up defluoridation plants or training for home based defluoridation techniques in study villages, should be considered.

## Background

Fluorosis is one of the severe public health problems in India, as almost two-third states are fluoride endemic [1]. In India, approximately 25 million people are presently affected by fluorosis and 66 million are at risk of developing fluorosis, including children of age 14 years [2]. India is situated in the geographical fluoride belt and in areas where fluoride content is high in rocks or soil, leaching of fluoride occurs, causing excess fluoride level in groundwater. However, the level of fluoride in water also depends on the natural solubility, presence of other minerals, the acidity of the soil and amount of water present, which explains high fluoride content in the groundwater [3].

Drinking water is the prime dietary source of fluorides. In addition, fluoride can also be present in foods such as salt water fish, sorghum, finger millets and crops grown in soil irrigated by water containing a high concentration of fluorides [4, 5]. Though, fluoride is an essential element for bone and teeth development as it forms hydroxyapatite with the calcium present in them and approximately 99% of the fluoride is found in calcified tissues of human body [5], and optimum fluoride level in diet prevent from dental caries, but high fluoride level exposures for a prolonged period results in dental fluorosis, skeletal fluorosis, and decrease in intelligence quotient [5–8].

Karnataka is one of the fluoride endemic states in India and 13 districts, including Kolar district, located in the eastern and south-eastern belt of Karnataka have reportedly high level of fluoride in the groundwater. However, limited studies have been done on the burden of dental fluorosis among these districts of Karnataka. Therefore, a school-based cross-sectional study was undertaken to quantify the prevalence and severity of dental fluorosis and to find the factors associated with dental fluorosis among the school going adolescents.

## Methods

### Study area

The present study was conducted in Kolar taluka, situated (N 13.1357, E 78.1326) in Kolar district on the border of three states *viz.* Andhra Pradesh, Tamil Nadu and Karnataka. Kolar district is situated in the eastern dry agro-climatic zone. It has a semi-arid climate with typical tropical monsoon followed by mild winters and hot summers. The predominant crops grown are finger millet (Ragi), groundnut and pulses. Finger millet, which is major staple diet of the people, occupies about 45% of the total cultivated area.

### Study design and sample size

A school based cross-sectional study was undertaken between the months of February and August 2013, among school going adolescents (age group 12–17 years) from randomly selected government and private schools in urban and rural areas of Kolar taluka. Sample size was calculated using prevalence of 32.6% [9] and absolute precision of 4%. Design effect of 2 was considered to arrive at a sample size of 1044.

A total of 55 schools were in the study area, among them 3 were only boys or girls school. Hence, 52 schools were considered for sampling (Urban area- 12; rural area — 40)

According to the cluster sampling technique and proportional allocation method, five schools from rural and one school from urban was selected by random sampling method and lottery method. All the high school children who were residents of the area since birth were included in the study.

### Sampling technique

Stratified cluster sampling was done according to the rural and urban setting and proportion allocation was performed to obtain the requisite number of schools. The selected schools were visited and classes were randomly selected, all students in a class were administered the questionnaire post informed consent from parents/guardians, and examination was done on the following visit. The primary inclusion criteria for study enrollment were students in the age group of 12–17 years, not suffering from any systemic illness.

### Data collection

#### Demographic and clinical data

A modified self-administered pretested questionnaire of WHO oral health assessment form [10] was piloted and subsequently used in the study. The study questionnaire was divided into two parts: first comprised of socio-demographic features, types of school and years of residence; second part mainly focused on individual details such as aids for oral hygiene maintenance, use of fluoridated or non-fluoridated toothpaste, details of drinking water source, consumption of finger millets, tea, and their intake frequency. A dental examination was done by dental specialist using a probe and mouth mirror in bright daylight with subject seated on an ordinary chair. Dean’s fluorosis index (DFI) was calculated based on the defined criteria [10, 11]. Severity and prevalence of dental fluorosis were assessed with DFI, and Community Fluorosis index (CFI) [12] was used to measure the burden of dental fluorosis in the study area.

#### Water samples

A total of 20 samples of groundwater, used for drinking purpose were collected from various sources around the school and of the village. Drinking water was collected from various sources in the village in doubly rinsed one liter sterilized plastic bottles and sent to Regional Occupational Health Centre (ROHC), Bangalore, where it was tested for fluoride level with the Orion method (Selective Electrode fluoride estimation apparatus) [13].

### Data analysis

Study data was entered twice in Epidata version 3.1 and statistical analysis was performed in Statistical Package for Social Sciences (SPSS) version 20 (IBM Corp, New York). Chi-square test was done to assess the association between water fluoride level and confounders such as fluoridated toothpaste, consumption of finger millet and tea. Karl Pearson test was performed to measure the association between fluoride content of drinking water and CFI. Multivariable analysis with generalized estimating equation (GEE) regression model was built to explore the association of independent factors with prevalence of dental fluorosis. GEE model was used to address the clustered data as recruited school children belonged to same cluster/school. “Presence of fluorosis in the participant” (Binary variable) was dependent variable in the GEE model. Independent variables were age group, gender, year of residence, type of school, ragi consumption, and drinking water source. Possible interaction between year of residence and water source (Shown as year of residence*water source) was also included in the GEE model. *P* value <0.05 was statistically significant.

## Results

**Fig. 1:**
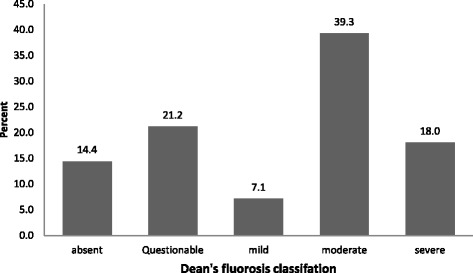
Dental fluorosis prevalence and severity among school going adolescents classified according to Dean’s Fluorosis index

A total of 1026 adolescent students of age group 12–17 years were included in the study. Majority of students were 15–17 years of age (54.9%) followed by 12–14 years (45.1%) of age group. The study population comprised of almost half female students (50.4%) and two third of total study participants were studying in Government schools (75.3%). A major number of participants were permanent residents of the particular village, residing in the same village either since birth or more than 10 years (96.3%). Majority of students had a mixed diet (94.4%) and tea consumption habit (86%). Finger millet was one of the major staple food (96%) as shown in Table 1. Figure 1 shows Dental fluorosis prevalence and severity classified according to DFI; moderate fluorosis was most commonly reported type of dental fluorosis among the participants.

**Table 1:** Socio demographic characteristics of study population (*n* = 1026)

Variables	Category	Frequency (%)
Age (in years)	Mean(SD)	14.7(1.01)
Age group	12–14 years	463 (45.1)
	15–17 years	563 (54.9)
Gender	Male	509 (49.6)
	Female	517 (50.4)
Monthly family Income	Less than Rs. 5000	434 (42.3)
	Rs. 5000–10000	488 (47.6)
	More than Rs. 10000	104 (10.2)
Type of school	Government	773 (75.3)
	Aided	253 (24.7)
Classes	8^th^	363 (35.4)
	9^th^	318 (31)
	10^th^	345 (33.6)
Diet	Vegetarian	57 (5.6)
	Mixed	969 (94.4)
Tea Consumption	Yes	887 (86.5)
	No	139 (13.5)
Finger millet Consumption	Yes	986 (96.1)
	No	40 (3.9)
Duration of residence	Less than 5 years	79 (7.7)
	5–10 years	38 (3.7)
	More than 10 years	58 (5.7)
	Since birth	851 (82.9)

Table 2 shows the prevalence of dental fluorosis was more than 70% among students residing in Beglihosahalli, Muduvadi, and Kurugal village. Other three study villages had 39.9–49.5% of students diagnosed with dental fluorosis. Mean fluoride content in water sample of five out of six villages was more than 1.2 gm/L, except Holur where it was 0.85 gm/L. Community fluoride index (CFI) was further calculated to assess the endemicity of fluorosis in the particular village and finding relevant public health significance.

**Table 2:** Village-wise prevalence of dental fluorosis and Community Fluorosis index

Village	Total Population^a^	Presence of fluorosis *n* (%)	Total students *n* (%)	Mean fluoride level in water (mg/l)	Community fluorosis index	Public health significance
Beglihosahallli	1292	214 (73.0)	293	2	2.8	Marked
Muduvadi	1449	157 (74.1)	212	1.6	2.12	Marked
Holur	2706	52 (49.5)	105	0.85	2.03	Marked
Sugutur	3421	31 (51.7)	60	1.4	2.3	Marked
Vokkaleri	3571	77 (39.9)	193	1.5	1.8	Medium
Kurugal	1864	131 (80.4)	163	1.2	2.59	Marked
Karl Pearson correlation coefficient						
6 villages	14303	662 (64.5)	1026	1.4 ± 0.38	2.3 ± 0.37	0.57

^a^According to Census 2011

Participation rate was more than 98% in all the villages

Table 3 shows GEE for multivariate analysis between individual determinants with the presence of fluorosis in the study population. Students who were residents of the study area and village by birth or more than 5 years were mostly affected by dental fluorosis compared to those living in endemic areas for less than 5 years (*p* < 0.001). Both male and female adolescents had almost equal prevalence of dental fluorosis. Adolescents from government schools had significantly higher dental fluorosis than private aided schools (71.1% vs 42.7%, *p* < 0.001).

**Table 3:** Generalized estimated equation (GEE) multivariable model of factors associated with prevalence of dental fluorosis (*n* = 1026)

Variables	Category	Fluorosis present	Total	β estimate (95%CI)	*P* value
Age group	12–14	294(63.5%)	463	−0.105(−0.379, 0.170)	0.454
	15–17	367(65.2%)	563	0	
Year of residence	<5 years	43(54.4%)	79	0.466 (−0.703,1.63)	0.435
	5–10 years	28(73.7%)	38	−25.3(−23.12,522.28)	<0.001
	Since birth	590(64.9%)	909	0	
Gender	Male	332 (65.2%)	509	−0.15(−0.42,0.12)	0.274
	Female	329 (63.6%)	517	0	
Type of school	Government	554 (71.7%)	773	−1.22(−1.53,-0.91)	<0.001
	Aided	108 (42. 7%)	253	0	
Ragi consumption	Yes	634 (64.3%)	986	0.241(−0.48,0.96)	0.509
	No	28(70.0%)	40	0	
Drinking water source	Bore well water	551 (63.7%)	865	0.92(−0.32,2.16)	0.145
	Pipe/tape water	79 (64.8%)	122	0	
Year of residence ^b^ Water source^a^	1^b^1			−0.464(0.82, 0.501)	0.479
	1^b^2			0	
	2^b^1			24.85(22.52,27.12)	<0.001
	2^b^2			0	
	2^b^3			0	
	3^b^1			0	

Dependent variable- Presence of fluorosis in the participant (Binary variable)

^a^Interaction variable- Year of residence ^b^Water source

Goodness of fit-Quasi Likelihood under Independence Model Criterion (QIC)- 1283.32

*P* value less than 0.05 considered statistically significant

*Abbreviation*: *Std error* standard error

## Discussion

Research studies have been conducted for estimating prevalence of dental fluorosis among school children in many districts of Karnataka state [6, 7, 9, 14], which lies in endemic fluoride belt in India, but the present study is one of the first in Kolar taluka. Dental fluorosis is a discoloration of teeth that is the most feasible indicator reflecting intake of higher than necessary level of fluorides, mainly through drinking water. Constant exposure to fluorides may lead to skeletal fluorosis and other serious consequences such as poisoning incidents including death. The study contributes to knowledge of burden of fluorosis in the area and to gain evidence for creating policies to regulate fluoride content of drinking water to an optimum level.

### Prevalence of dental fluorosis

Fluoride uptake by the enamel occurs during the development of enamel i.e. mineralization stage. Dental mottling usually occurs in permanent teeth and is visible clearly in age groups of 5 years and above. Therefore, the prevalence of dental fluorosis among the children of growing age is considered as the expression of the current problem of fluorosis. Our study results showed that nearly two third of participants had experienced dental fluorosis. Similar studies conducted in different parts of India reported the prevalence as 66.7, 71.3 and 69.3% among school children [2, 15, 16]. On the other hand, a comparatively lower prevalence of 32.6 and 15.8% was recorded in Kerala and Tamil Nadu respectively [9, 17]. These differences of prevalence in dental fluorosis were mainly due to fluoride content in the water available in the area. Studies from China and Brazil have detected high fluoride prevalence ranging from 50 to 80.4%, the results which are similar to present study [18, 19].

### Gender differences and category of fluorosis

Whilst, a higher prevalence of dental fluorosis among females was reported in a few studies [8, 20] and one study reported a higher prevalence among the males [9], the present study did not find any significant difference in burden of fluorosis between the sexes. Our study findings are consistent with research conducted among rural school children in Uttar Pradesh [21]. A study conducted in 2009 among primary school children in Chikkaballapur district of Karnataka [20], showed an increased prevalence of very mild dental fluorosis, according to the Dean’s Fluorosis Index when compared with other categories. Our study suggests highest prevalence of moderate fluorosis which was 39.3 percent among participants.

### Factors associated with dental fluorosis

The present study examined predictors of dental fluorosis among the participants. The significantly related factors to dental fluorosis were living in the study area for more than 5 years and studying in government schools, which in turn were related to low socio-economic status. Fluorosis affects the tooth during developmental stages, which is a plausible explanation to the fact that it occurred in children living in the area since birth or more than 5 years. According to the present study, occurrence of dental fluorosis was similar among children who consumed water from the bore well (63.7%) *or* tap via pipe supply (64.8%). A study done by Gopalakrishnan et al. in Kerala [8] reported prevalence of dental fluorosis was significantly higher among children who consumed pipe water (44.8%) as compared to children who consumed well water (12.7%). The high fluoride content of the drinking water available from CFI data was positively correlated with dental fluorosis indicating consumption of drinking water to be related with high dental fluorosis among the participants.

### Water fluoride content and community fluorosis index

The WHO guideline for the maximum permissible limit of fluoride in drinking water is 1.5 mg/L, higher concentrations of fluoride can cause fluorosis of varying degrees. Bureau of Indian standards has further reduced the safer limit of fluoride intake to 1.1 mg/L. The mean value of the fluoride content, among six villages, was 1.4 with a standard deviation of 0.38 whereas the maximum and minimum fluoride content, in samples collected at various locations in the village, was found to be 2.0 and 0.4 mg/L respectively. More than permissible limits of fluoride content in drinking water in the study area point towards risk of deleterious effects among the population due to chronic fluoride exposure.

The present study reported a Community Fluorosis Index of 2.3 which is very high in comparison to CFI of 0.59–0.79 reported in two Chilean cities [22] and Ramnagaram district, Karnataka [23], where it was reported as 1.76. A significant positive correlation was observed between CFI and fluoride content of drinking water (rho = 0.570), which reinforces the fact that villages with a higher level of fluoride have higher Community Fluorosis Index. Several other studies have similar findings for correlation between CFI and fluoride content of drinking water [1, 13, 14, 24].

This study indicates a need for further research for identifying more determinants associated with dental fluorosis which can be beneficial in developing strategies for appropriate intervention. In addition, deflouridation of drinking water and its pipeline supply has to be undertaken for water sources where the fluoride content is more than the permissible limits to make the water risk-free in future.

### Limitation

The limitation of this study was only oral examination was done to report dental fluorosis (as it is a convenient biomarker), but skeleton fluorosis examination and invasive procedures like fluoride level estimation of blood and urine were not undertaken. Skeletal fluorosis helps to detect long-term effect of fluoride exposure. Furthermore, finding all associated factors related with fluorosis were not within the scope of research.

## Conclusion

Although dental fluorosis is an irreversible condition of the enamel of teeth, but can be prevented if the level of fluoride in water is optimum. Regular water testing, routine medical check-up camps, and continued health awareness program would certainly benefit the community residing in fluoride endemic areas. Additionally, the study had attempted to bring the attention of policymakers towards the magnitude of the condition and to implement strategies such as setting-up of community defluoridation plants or training on home based indigenous defluoridation techniques so that fluoride content of drinking water at village or community level can be optimized and subsequently regulated.

## Abbreviations

CFI: Community fluorosis index
DFI: Dean’s fluorosis index
GEE: Generalized estimating equation
ROHC: Regional occupational health centre
